# An injectable nucleus pulposus cell-modified decellularized scaffold: biocompatible material for prevention of disc degeneration

**DOI:** 10.18632/oncotarget.16831

**Published:** 2017-04-04

**Authors:** Zhi Shan, Xianfeng Lin, Shengyu Wang, Xuyang Zhang, Yichuan Pang, Shengyun Li, Tianming Yu, Shunwu Fan, Fengdong Zhao

**Affiliations:** ^1^ Department of Orthopaedic Surgery, Sir Run Run Shaw Hospital, Medical College of Zhejiang University, Hangzhou, 310016, China; ^2^ MOE Key Laboratory of Macromolecular synthesis and Functionalization, Department of Polymer Science and Engineering, Zhejiang University, Hangzhou, 310027, China

**Keywords:** intervertebral disc degeneration, small intestinal submucosa, nucleus pulposus cell, decellularization, extracellular matrix

## Abstract

We developed a nucleus pulposus cell (NPC)-modulated decellularized small intestinal submucosa (SIS) scaffold, and assessed the ability of this material to prevent Intervertebral disc degeneration (IVD) degeneration. Decellularized porcine SIS was squashed into particles and the biological safety and efficiency of these particles were evaluated. Next, SIS particles were seeded with rabbit NPCs, cultured for two months *in vitro*, decellularized again and suspended for intervertebral injection. We demonstrated that use of the decellularization protocol resulted in the removal of cellular components with maximal retention of extracellular matrix. The xenogeneic decellularized SIS did not display cytotoxicity *in vitro* and its application prevented NPC degradation. Furthermore, the xenogeneic SIS microparticles were effective in preventing IVD progression *in vivo* in a rabbit disc degeneration model. In conclusion, our study describes an optimized method for decellularized SIS preparation and demonstrated that the material is safe and effective for treating IVD degeneration.

## INTRODUCTION

Low back pain is common in the elderly population, with a population prevalence of 15–30%, and a lifetime prevalence of over 60%. [1] Intervertebral disc degeneration (IDD) is believed to be the leading force of Low back pain by changing the biomechanical situation of the spine.[2] Also, the IDD can generate discogenic pain directly. [3] Low back pain associated with IDD can progress to multiple spinal disorders, including disc herniation, spondylolisthesis, and spinal stenosis. [4] Although there are many operative and non-operative approaches for treating IVD degeneration, such as pain control and discectomy of spinal fusion, most only address the symptoms and do not restore the natural structure of IVD.

Regeneration of degenerated disc tissues could restore IVD structure and treat IDD. While a proper extracellular matrix (ECM) could contribute to the regeneration of organs and tissues, decellularization is an attractive technique for preparing an ECM scaffold for tissue repair. Physical (shocks and freeze/thaw cycles), enzymatic (DNase and trypsin), and chemical (Triton X-100 and sodium dodecyl sulfate) protocols can achieve the decellularization of tissues and enable the acquisition of a cell-free ECM. [5] By removing the allogeneic or xenogeneic cellular components, the immunogenic response towards an ECM scaffold can be reduced while preserving most of the biomechanical and biological properties. Several studies have shown the potential of decellularization for the regeneration of IVD. Simionescu and Xu *et al*. reported the decellularization of native-derived nucleus pulposus (NP) and annulus fibrosus (AF) tissues separately and demonstrated the biological compatibility of the material. [6–8] Cheung *et al*. [9] decellularized a whole IVD and removed 70% of the endogenous cells within the disc. Chan *et al*. considered collagen I to be the main component of NP ECM and obtained decellularized NP. [10] These studies show that decellularization is a feasible approach to obtain material for preparation of an ECM scaffold.

**Figure 1:**
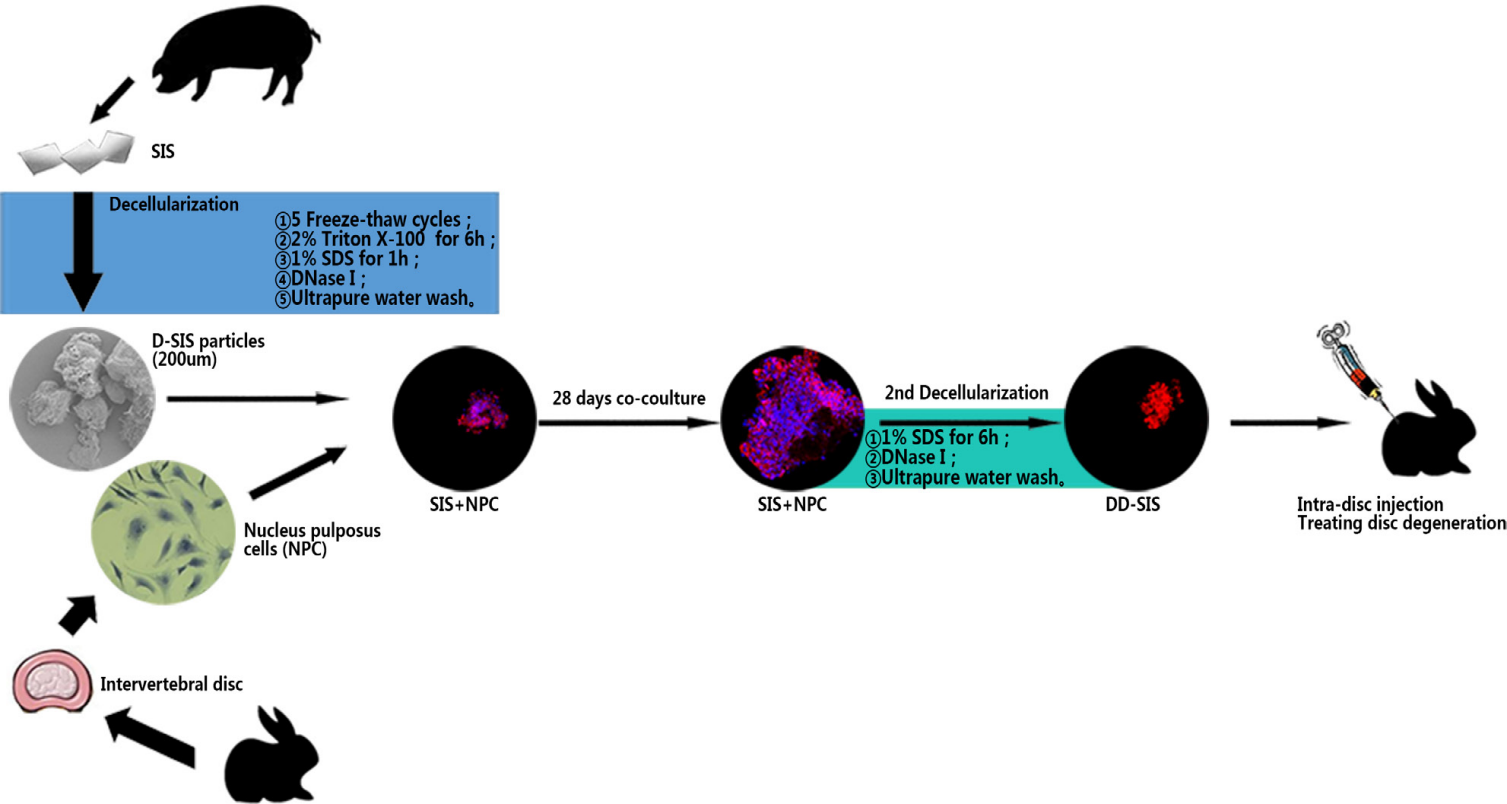
Schematic representation of the overall research Abbreviations: SIS = small intestinal submucosa; D-SIS = decellularized SIS; DD-SIS = second decellularized SIS; NPC = nucleus pulposus cells.

Decellularized NP has limitations when used as a scaffold, as the decellularization process can damage NP structure and bioactivity. [11] In addition, the NP is relatively difficult to acquire. SIS and NP share part of the ECM[12, 13], but when compared with NP materials, SIS is more economical, more convenient to acquire, and has a tougher structure. Thus, SIS could be an ideal scaffold material for regenerating IVD. As many researches supports that NP degeneration is a major part in disc degeneration process, the current study focused in retarding the NP degeneration [14]. Here, we used decellularized SIS (D-SIS) particles as a scaffold, used NPCs to modify the ECM of SIS, decellularized the SIS again (DD-SIS), and used the latter material to treat disc degeneration. A schematic outline of the study is depicted in Figure 1.

Porcine-derived SIS from small intestinal tissue applied as a completely decellularized matrix was first described as a vascular substitute. [15, 16] Research has shown that decellularized SIS contains bioactive factors such as fibronectin, glycosaminoglycan (GAG), and growth factors, [17] as well as bioactive factors involved in cell adhesion, mitogenesis, and chemotaxis. [18] Ligament reconstruction using SIS was demonstrated to be effective in equine, caprine, and canine models. [19–22] Badylak *et al*. introduced SIS for Achilles tendon reconstruction in a dog model. [23] In a rodent model of abdominal wall defection, SIS displayed the ability to restore functional skeletal muscle structure. [24] Also, SIS has been proved to be useful in hernia repair, [25] Esophageal preservation, [26] skin replacement and wound repair in human. [27]

## RESULTS

### Optimized decellularized method for SIS

**Figure 2:**
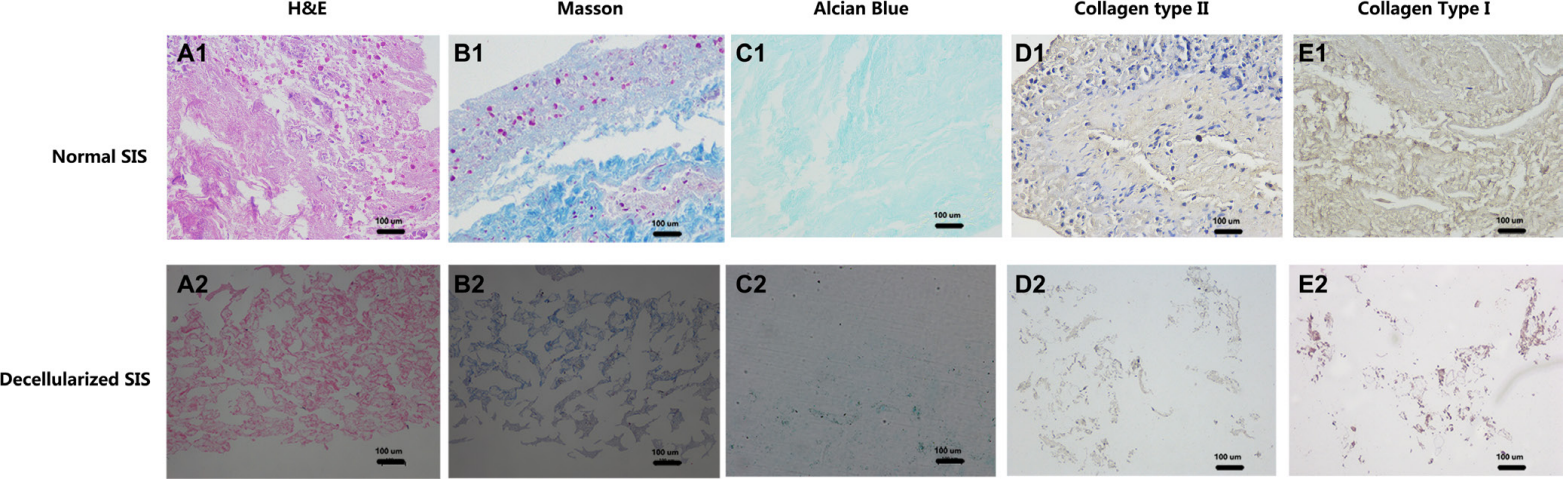
Effects of first time decellularization Histological images of normal SIS (**A1–E1**) and decellularized SIS (**A2–E2**) confirmed the effect of decellularization, that no nuclear material can be found after decellularization, while part of collagen and aggrecan are maintained. Abbreviations: SIS = small intestinal submucosa.

**Figure 3:**
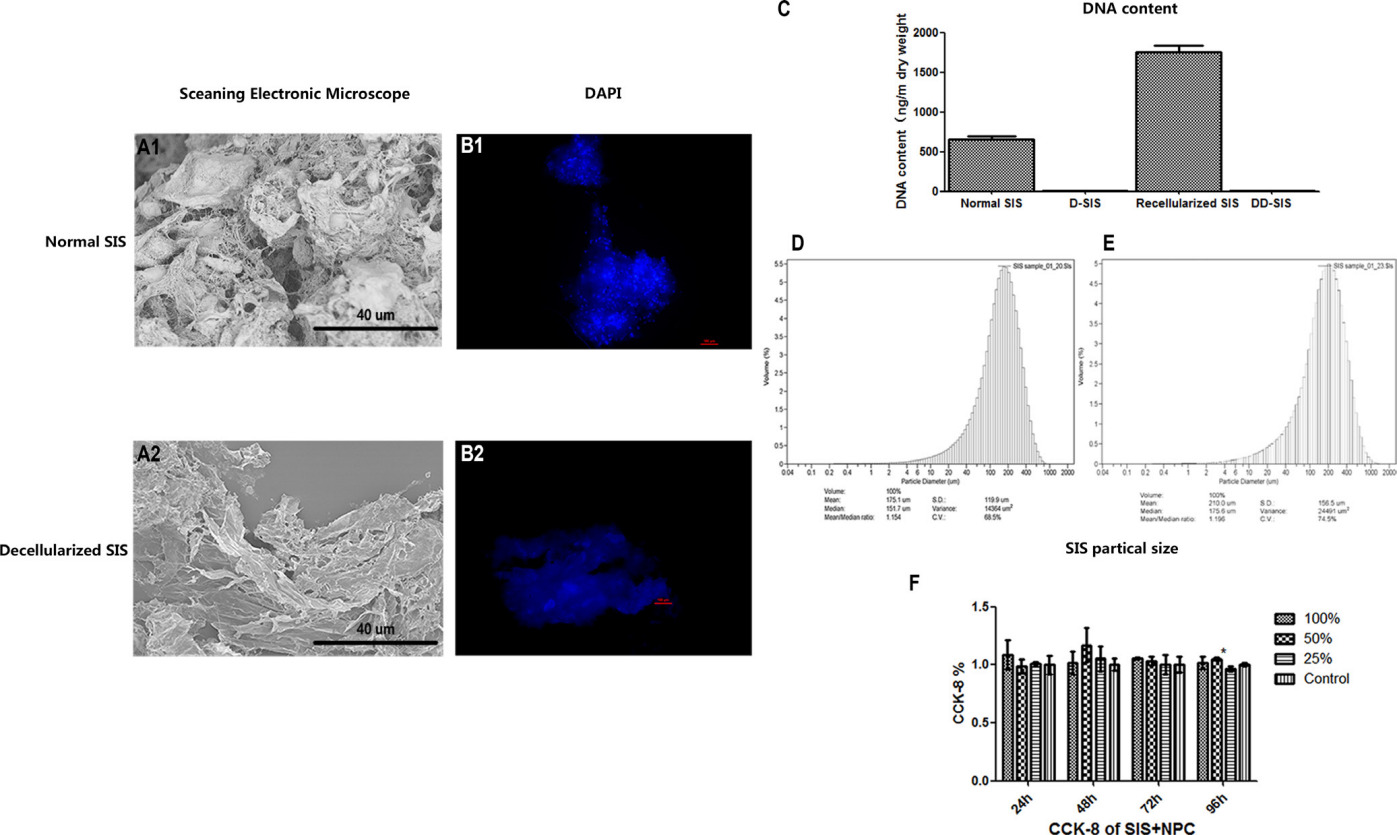
Effects of first time decellularization and SIS particle analysis Scanning electronic microscope image showing that that the SIS ultrastructure was maintained (**A**), fluorescent images (**B**) and DNA content (**C**) of normal SIS, decellularized SIS, SIS recellularized with NPC and DD-SIS confirmed the efficiency of decellularization is good. SIS particle size was approximately 200 μm after 1^st^ (**D**) and 2^nd^ (**E**) time decellularization. CCK-8 demonstrated good cytocompatibility (**F**). Results (C and F) are represented as mean +/− SEM. Abbreviations: SIS = small intestinal submucosa; DAPI = 4,6-diamidino-2-phenylindole, CCK-8 = cell counting kit-8.

**Figure 4:**
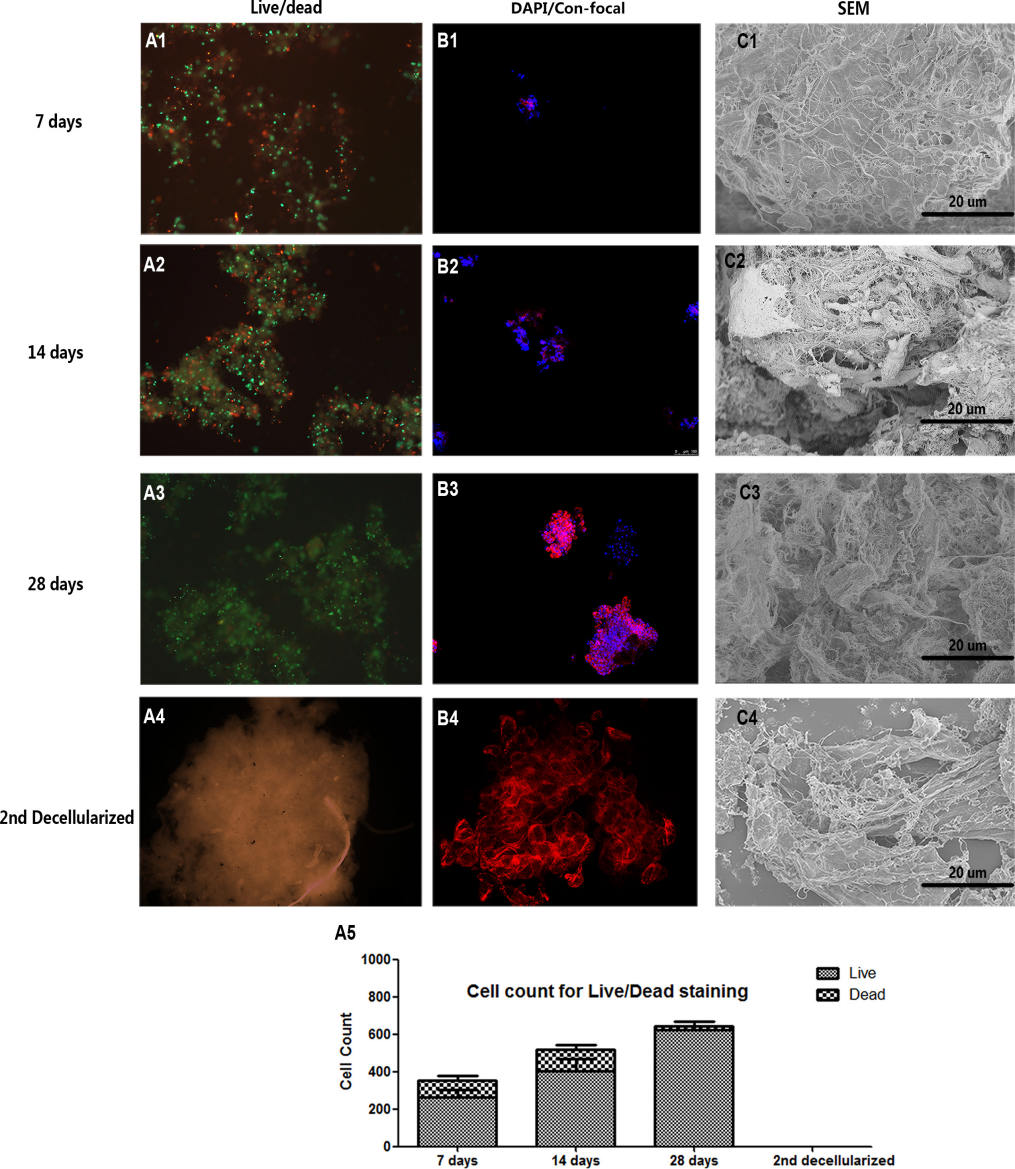
Decellularized SIS support NPC proliferation *in vitro* Live-Dead cell staining (**A1**–**A3**), cell core confocal fluorescent images (**B1**-**B3**) and SEM (**C1–C3**) of NPC proliferation suggesting that decellularized SIS can support NPC proliferation generate ECM *in vitro*. After the second decellularization, all cell components were removed (**A4–C4**). The cell count for Live-Dead staining of each time point is shown in (**A5**). Abbreviations: SEM = small intestinal submucosa; DAPI = 4,6-diamidino-2-phenylindole.

After the preparation of D-SIS and DD-SIS, H&E staining was performed to evaluate the overall effect of the decellularization process. As presented in Figure 2A1 and 2A2, SIS presented pink eosinophilic staining indicating collagen. The effect of decellularization can also be confirmed by Masson staining (B1, B2). In contrast, no basophilic staining indicating nuclear material was observed in decellularized SIS (Figure 2A2). Alcian blue staining (Figure 2C1 and 2C2) showed that GAG content was partly preserved after decellularization to generate a suitable environment for NP cells. Immunohistochemical staining revealed that collagen type I and type II content was also preserved after decellularization. The degree of DNA decreased from 653.18 ng/mg before to 3.81 ng/mg following decellularization. SEM analysis revealed complex fiber networks in the D-SIS, indicating that the SIS ultrastructure was maintained (Figure 3A). D-SIS particle size was approximately 200 μm in diameter after 1st and 2nd time decellularization, a suitable size for cell anchorage (Figure 3C). At the second decellularization, a milder protocol was used. H&E staining, immunohistochemical staining, and DAPI staining showed complete removal of the cells, with preservation of the ECM (Figure 4C4). Together, these data suggested that the decellularization strategy resulted in removal of the cellular components while preserving ECM content and structure.

### Decellularized SIS supports NPC proliferation *in vitro*

**Figure 5:**
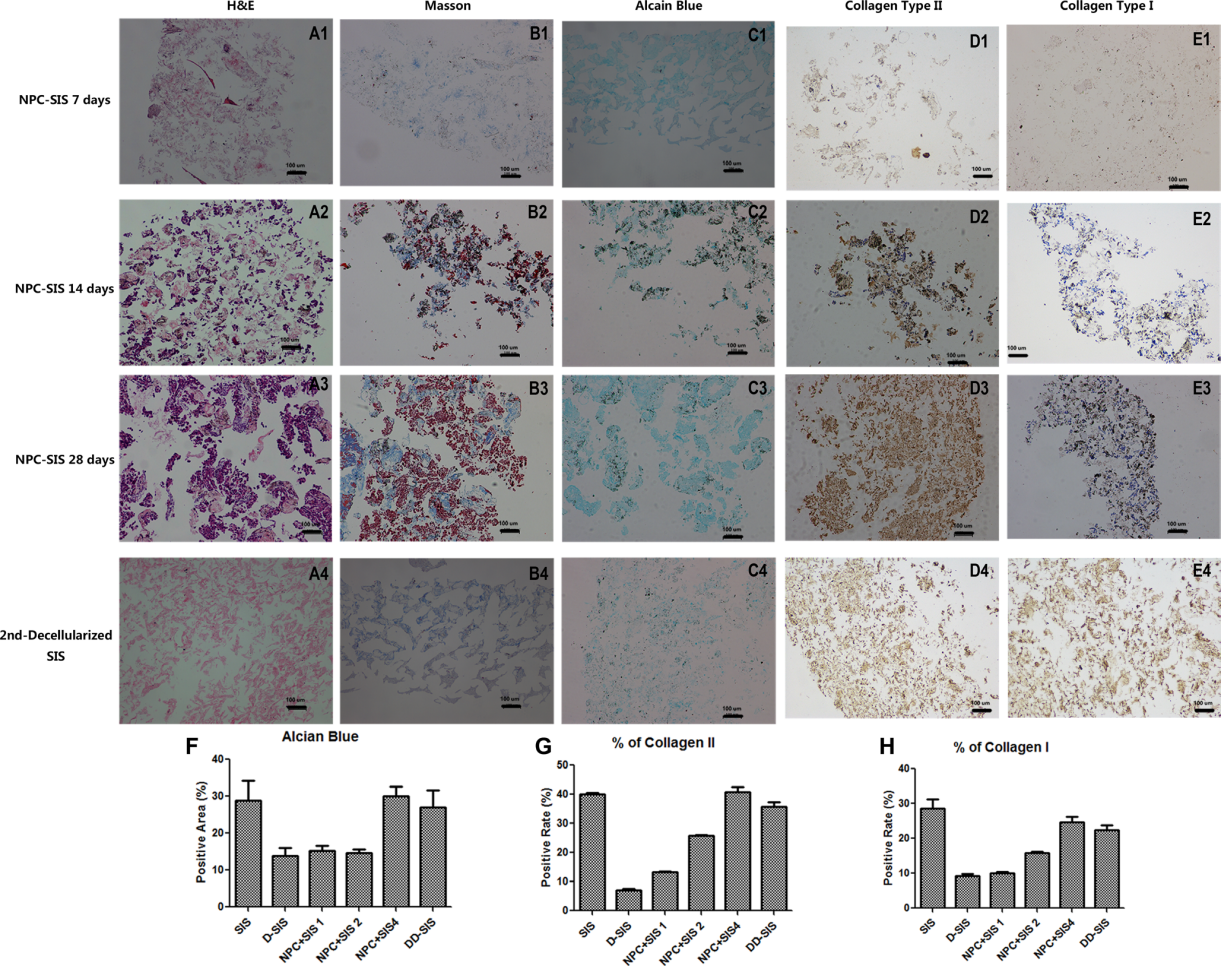
Decellularized SIS support NPC function *in vitro* H&E (**A1-D1**), Masson (**A2-D2**), Alcian blue (**A3-D3**), collagen type II (**A4-D4**) and collagen type I (**A5-D5**) staining suggesting that decellularized SIS can support NPC proliferation and generate ECM *in vitro*. After the second decellularization, all cell components were removed (**E1-E5**). Quantitative analysis showed suggesting that decellularized SIS can support NPC proliferation and generate Collagen II and GAG *in vitro*, and the second time of decellularization didn't damage the ECM (F–H). Results (**F**, **G** and **H**) are represented as mean +/− SEM, and normalized to allow the exclusion of the blank areas. Abbreviations: SIS = small intestinal submucosa.

To modify the D-SIS to make it more suitable for IVD reconstruction, we repopulated NPCs onto the D-SIS suspension and evaluated the material *in vitro*. Live-Dead cell staining showed the long-term viability of NPCs after being seeded for 7, 14 and 28 days. At each time point, live cells (stained green) occupied the majority of the area (Figure 4A). As time progressed, more live cells were observed in the inner region of the D-SIS particles. H&E staining confirmed that NPCs attached and proliferated with D-SIS (Figure 5A). DAPI staining also showed that cell distribution was in agreement with the above results (Figure 4B). Furthermore, we evaluated the morphology of these repopulated SIS using SEM, which revealed the presence of column, strip-like NPCs anchored on the SIS particles at day 14. At day 28, cells maintained a strip-like shape, which likely contributed to the maintenance of NPC morphological structure *in vivo* (Figure 4C).

### Decellularized SIS supports NPC synthesis function *in vitro*

**Figure 6:**
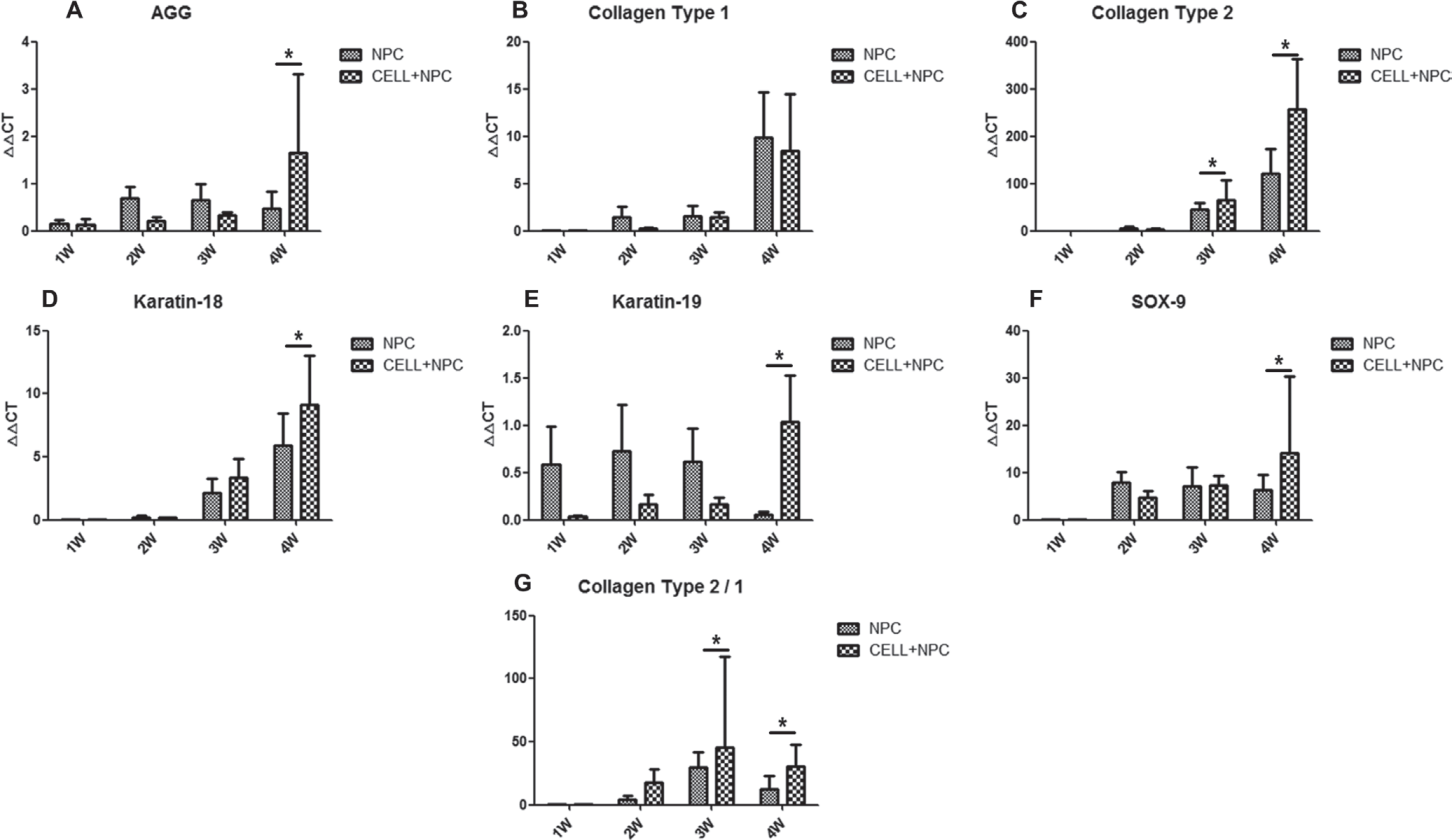
IVD-related gene expression in NPCs seeded in D-SIS evaluated by RT-PCR Gene expression data are normalized to the average number of primary nucleus pulposus cells. Expression of aggrecan, collagen type I, collagen type II, keratin-18, keratin-19 and SOX-9 were significantly up-regulated at day 21 (**A–F**), the ratio of collagen type II/collagen type I was significantly greater in the SIS co-culture group (**G**). Data are represented as mean +/– SEM. Abbreviations: AGG = aggrecan; COL-1 = collagen type I; COL2 = collagen type II. * indicates statistical significant difference.

To investigate the synthesis function of SIS repopulated with cells, we compared the expression of NP-related genes between NPCs seeded with D-SIS and NPCs cultured on a plate. RT-PCR results showed that when seeded in an SIS suspension, expression of genes encoding collagen type II, type I, and aggrecan increased after the first week and remained stable at day 21. Conversely, expression of these factors in NPCs cultured alone on a plate progressively decreased during the same period. At day 28, expression of aggrecan, collagen type II, keratin-18 and keratin-19 were 3.5-, 2-, 1.5- and 17-fold higher than that of the control group, respectively. Expression of the gene encoding SOX-9, a typical IVD cell marker, was 2.5 times greater than that in cells cultured alone. The ratio of collagen type II to collagen type I was also significantly greater in the SIS co-culture group. RT-PCR results are presented in Figure 6. In all, these results suggested D-SIS supported NPC survival and function *in vitro*.

### Decellularized xenogeneic DD-SIS prevented disc degeneration *in vivo*

**Figure 7:**
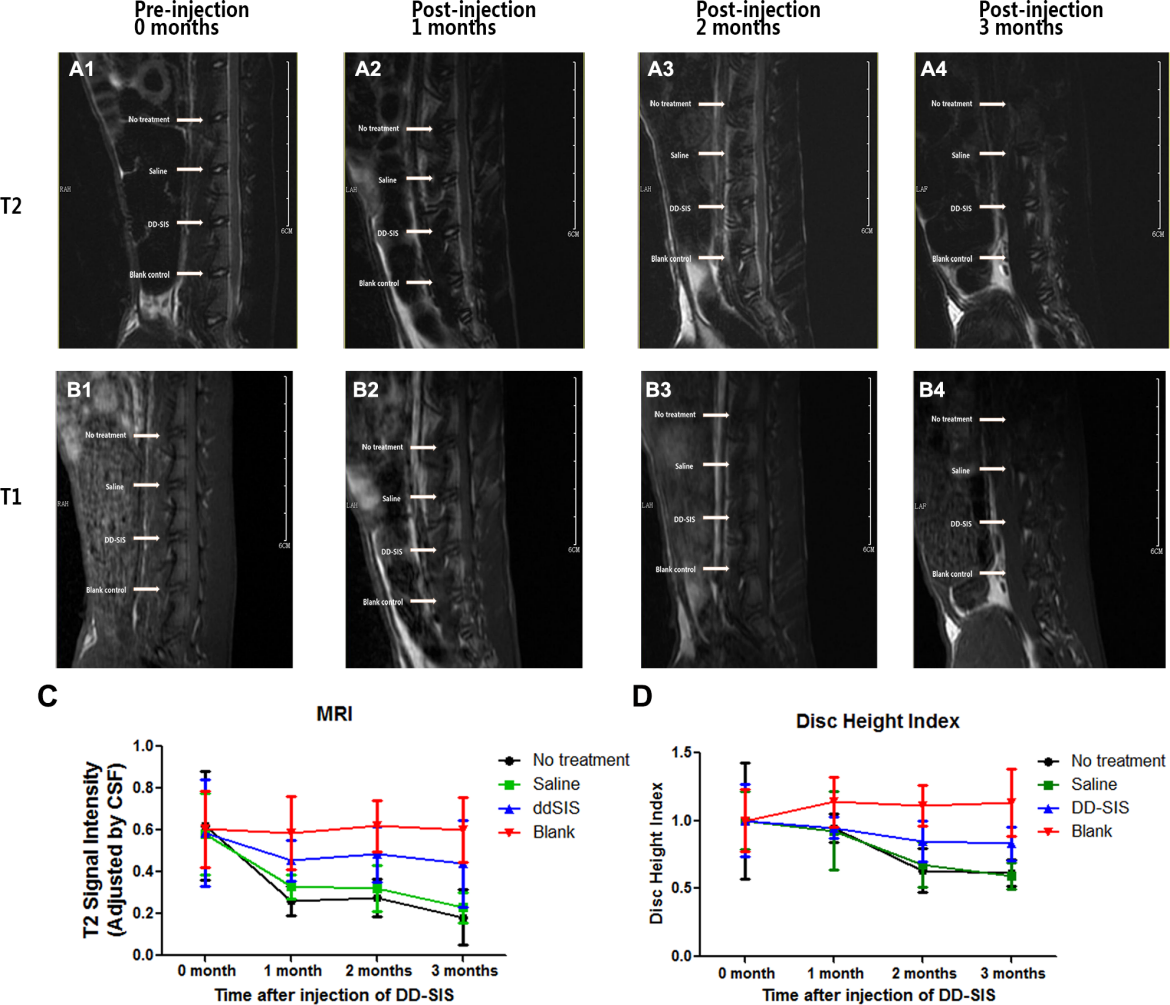
DD-SIS prevents disc degeneration *in vivo* Representative continuous and dynamic changes of IVD revealed by magnetic resonance imaging (MRI) on T2-weighted **(A)** and T1-weighted **(B)** images. Quantitate analysis **(C)** of T2 disc signal intensity showed that DD-SIS prevents disc degeneration *in vivo*. Disc height index indicated by X-ray at different time points **(D)** showed better preserved disc height in DD-SIS group. *n* = 8 per group per time point. Abbreviations: SIS = small intestinal submucosa; DD-SIS = second decellularized SIS.

Given its ability to support NPCs *in vitro*, the effects of DD-SIS were further evaluated *in vivo* using a rabbit IDD model. Representative MRI and X-ray images from each time point are presented in Figure 7. The signal intensity of IVD injected with saline showed a decreasing trend with time, while the IVD injected with DD-SIS displayed significantly higher signal intensity at 2 and 3 months (*P* < 0.001, Figure 7A, 7B). When evaluated by X-ray, disc height in the DD-SIS-treated group was larger than that in the saline-treated group, and the differences were significant between DD-SIS group and Saline/No treatment group at 2 and 3 months (*P* < 0.001, Figure 7C).

## DISCUSSION

In this study, we developed an optimized decellularization method that could successfully remove the cellular components of SIS while maintaining its structure. NPCs grow well when seeded to the D-SIS *in vitro* and can modify the microenvironment, making the SIS more suitable for IVD reconstruction. We demonstrated that intradisc injection of DD-SIS has a therapeutic effect in an animal model of IDD.

SIS is one of the most established and broadly applied biological matrices. This type of matrix has been previously used for cardiovascular, [28] urogenital, [29] gastrointestinal [30] and ligament [23] reconstruction tests *in vivo*. Yang *et al*. observed that SIS can stimulate bladder smooth muscle cells and human umbilical vein endothelial cells to attach, proliferate and migrate *in vitro*. [31] Li *et al*. showed that small molecular weight peptides released by SIS are biologically active with respect to recruitment of murine endothelial cells *in vitro* and *in vivo*. [32] Cell-seeded SIS has also been investigated. Du *et al*. reported a successful tracheal reconstruction in rabbits using SIS seeded with a monolayer of mesenchymal stem cells. [33] In this study, we firstly attempted to use NPC-modified SIS for treating intervertebral disc degeneration.

Since immune responses can facilitate IVD, decellularization was an essential step in our protocol. Because SIS is a dense tissue, we applied chemical treatments after physical treatment (snap-freeze/thaw cycles) to loosen the SIS tissue and remove the cellular antigens. The main bioactive components required for IVD regeneration, such as aggrecan, COL-I, and COL-II were well preserved upon both first and second decellularization processes. In addition, DNase was used, as insufficient removal of DNA fragments is associated with a greater risk of a pro-inflammatory or immune responses. [34, 35] All the washing steps of decellularization were carried at a low temperature (4°C) to inhibit proteases and maintain SIS ultrastructure for SEM visualization. Therefore, we developed an optimized SIS decellularization protocol for IVD regeneration.

For intervertebral discs, there is still no effective screening platform for regenerative drug development. Cells are an indispensable part of tissue regeneration therapy. We showed that expression of functional NP-specific factors decreased when NPCs were cultured in a standard environment, suggesting potential spontaneous NPC degeneration. These results are consistent with previous reports showing that chondrocytes display decreased proliferation and matrix synthesis during expanded culture. [36] Therefore, scaffolds that can support normal NPC proliferation and synthesis are essential. By seeding NPCs into decellularized SIS, we observed that NPC degeneration was inhibited. With the support of SIS, NPCs maintained their original column and strip-like shapes, and their synthesis ability was preserved, as shown by the NP-related gene expression results. By binding to promoters of genes encoding Col-II and aggrecan, SOX-9 is also essential in NPC proliferation, [37, 38] and expression of these factors was preserved when NPCs were cultured with SIS. We conclude that a preserved collagen-rich microstructure, together with bioactive proteins and growth factors supported by the SIS, play an important role in inhibiting NPC degeneration. These factors, acted as footprints, facilitating NPC attachment, proliferation, and protein synthesis. In addition, a particle size of approximately 200 μm was shown to be ideal for optimal cell adhesion [39, 40].

Being a widely-studied biomaterial, SIS has its technic and advantages. Also, the cost of NPC culturing and decellularization are acceptable. Thus, the economic feasibility of clinical usage of the DD-SIS is good. There were some limitations to our study. Firstly, we only tested a successful protocol for SIS decellularization and NPC culture. The effects of our protocols on different cell factors and signaling pathways associated with IVD regeneration were not explored in detail, and it requires further research. Previously articles have suggested that some growth factors, such as TGF-β, bone morphogenetic protein-2 and growth differentiation factor-5 could enhance the effect of intervertebral disc remodeling. [41–43] Secondly, we used injection of DD-SIS into discs of an animal model to treat IDD, while a needle puncture itself is an IDD risk factor. To reduce these negative effects, the needle size we used to inject DD-SIS was 28 g, which was much smaller than the size of the IVD. According to previous research, the puncture-induced degenerative changes can be largely affected by needle size. [44] Finally, a rabbit model with small IVD may be more favorable for regeneration, while this IVD degeneration model has been used in many researches and proved to be acceptable. Future research should focus on the mechanism of how SIS interacts with NPCs using proteomic and genetic approaches.

## MATERIALS AND METHODS

### Research overview

SIS samples were harvested from Bama miniature pigs aged two months and grinded into particles 100–300 μm in diameter. The cellular components were completely removed while the ECM structure of SIS was maintained. The determination of a successfully decellularized SIS was based on histological, DNA, and collagen content analyses. To evaluate the biological safety of xenogeneic SIS, the material was implanted into xenogeneic subcutaneous tissue *in vivo*. Rabbit NP cells were reseeded in decellularized SIS and cultured *in vitro*, and rabbit NPC growth was evaluated using histological, biochemical, real-time PCR, and electron microscopy. Next, after two months of co-culture, reseeded SIS was decellularized again, evaluated histologically, and injected *in vivo* to investigate its effects on treating of disc degeneration. Radiological evaluations of functional outcomes, such as disc height and degeneration level, were performed using X-ray and MRI analyses. All animal experiments were approved by the Animal Experimental Ethics Committee of Zhejiang University and animals were treated according to approved experimental protocols.

### Harvest and decellularization of SIS

SIS material was collected from Bama miniature pigs that were approximately two months old. Jejunum sections were harvested within 2 h of sacrifice and then prepared according to a previously published method. [45] To separate SIS from porcine jejunum, fat was first removed, followed by careful washing with water and a saline solution, and then cut into pieces 10 cm in length. Tunica serosa and tunica muscularis were carefully removed mechanically using gauze. An optimized decellularized protocol should completely remove the cellular components of SIS while fully maintaining the three-dimensional structure of the ECM. Prior to other decellularization procedures, five snap-freeze-thaw cycles (liquid nitrogen and 37°C water bath) were performed to facilitate the diffusion of decellularized reagents. After freeze-thaw processing, SIS particles were decellularized using 2% Triton X-100 (Sigma-Aldrich, St. Louis, MO, USA) for 6 h and SDS (1%, Sigma-Aldrich) for 1 h and next processed with DNase I (100 U/mL, Sigma-Aldrich, 37°C, 6 h). After decellularization, samples were washed in ultrapure water (2 days, 3 exchanges per day) and rinsed in PBS (1 day). All decellularization steps were performed by submerging the tissue in the decellularization reagents and incubating with agitation (120 rpm).

### Preparation of SIS particle scaffold

The SIS pieces after decellularization were pulverized into 100–200 μm diameter particles with a tissue homogenizer (Precellys 24, Bertin Technologies, Paris, France) in liquid nitrogen (6,000 rpm, 20 cycles, 30 s/cycle). After pulverization, a suspension was obtained and filtered through an 80-mesh cell sieve (200 μm), which controlled the size of harvested SIS. SIS particle sizes were tested using a Laser Particle Size Analyzer (LS13-320, Beckman Coulter, Brea, CA, USA). Next, SIS particles were immediately frozen in liquid nitrogen for later use.

### Isolation and culture of rabbit NP cells

Rabbit NPCs were isolated from the NP tissue of two month-old New Zealand white rabbits with type I collagenase (5%) and cultured in Dulbecco's modified Eagle medium with high glucose (DMEM-HG, Gibco, Grand Island, NY, USA) plus fetal bovine serum (10%, Gibco, Mulgrave, Australia), penicillin (100 U/mL), streptomycin (100 μg mL^-1^), and amphotericin B (2.5 μg mL^-1^, 37°C) in a humidified atmosphere that contained CO_2_ (5%). Cells were seeded in T75 flasks and allowed to adhere for 24 h. After three washes with PBS, adherent cells were cultured in complete DMEM-HG. Cells were detached using trypsin-EDTA (0.25%) upon 80–90% confluence and subcultured at passage 1 (P1). Cells at P2 were used for the recellularization for the D-SIS.

### Cytocompatibility testing of SIS (CCK-8)

SIS cubes were incubated in DMEM-HG medium for 48 h in a humidified atmosphere with CO_2_ (5%, 37°C) at and the leaching solution was collected for later use. NPCs at P2 were seeded in 96-well cell culture plates (5 × 10^3^ cells/well) in standard DMEM-HG (200 mL) for 24 h. The medium was then removed and replaced with the leaching solution (25%, 50% and 100%) and incubated for six days. At days 1, 2, 3, and 4, cell counting kit-8 (CCK-8) assay (Dojindo Laboratories, Kumamoto, Japan) was added to each well, and then the plate was incubated in the incubator (30 minutes).

### Repopulation of NP cells into an SIS particle scaffold

Research has shown that appropriate ECM can delay spontaneous senescence of chondrocytes. [46] Therefore, we seeded NPCs (7 × 10^6^ cells mL^-1^) into SIS particles suspended in DMEM-HG to evaluate its effect on degeneration. Cells from the same source were cultured with DMEM-HG (5 × 10^5^ cells mL^-1^) as a negative control.

### Cell viability and metabolic activity of recellularized SIS

After repopulation, a Live-Dead cell staining Kit (BioVision, Milpitas, CA, USA) was used to distinguish live and dead cells and the cellular activity of the recellularized SIS was observed at 1, 2, 3 and 4 weeks. Live NPCs (stained green) were visualized using fluorescence microscopy, counted and recorded as the mean of 10 randomly-selected views from each specimen at 10 × 10 magnification, and were compared with the positive control group (NPCs cultured in standard DMEM-HG) to determine the cytocompatibility of SIS particles.

### Evaluation of histological, histochemical, immunohistochemical and fluorescent staining

Normal SIS, D-SIS, recellularized SIS at 7, 14 and 28 days, and DD-SIS were washed with PBS three times, fixed in PFA (4%, 24 h), followed by standard paraffin embedding and sectioning. For *in vivo* treatment, the IVD were harvested from the rabbits three months after injection of DD-SIS, fixed in PFA (4%, 48 h), decalcified (30 days) and embedded in paraffin. Hematoxylin and eosin staining was performed to reveal the whole structure and cell distribution. Alcian blue staining was performed to evaluate retained GAG content. Specimens for immunohistochemistry were processed as previously described by our group. [47] Epitopes of interest included collagen type I (Abcam, Cambridge, MA, USA), collagen type II (Novus Biologicals, Littleton, CO, USA) and aggrecan (Novus Biologicals). 4,6-Diamidino-2-phenylindole (DAPI, Sigma) fluorescent nuclear staining was performed to evaluate the efficiency of decellularization and NPC distribution at different time points.

### DNA concentration analysis

Genomic DNA of SIS, D-SIS, recellularized SIS at different time points and DD-SIS was extracted and isolated using a DNeasy Blood & Tissue Kit (Qiagen, Hilden, Germany), according to the manufacturer's protocol. DNA concentration was measured by 260nm ultraviolet absorption using a NanoDrop 8000 instrument (Thermo Fisher Scientific, Wilmington, MA, USA).

### Scanning electron microscope

Scanning electron microscopy (SEM) was performed to examine the microarchitecture of D-SIS and recellularized SIS. Particles were washed with ddH_2_O, freeze-dried, sputter-coated with gold-palladium and viewed under the SEM (SU8010, Hitachi, Japan).

### Gene expression of IVD cell markers by RT-PCR

The mRNA from NPCs-seeded SIS was extracted with RNeasy Mini Kit (Qiagen, Valencia, CA, USA) according to the manufacturer's instructions. RNA integrity and concentration were determined using a NanoDrop 8000 instrument. Reverse transcription was performed at 45°C for 50 minutes and 82°C for 5 minutes using 5× Prime Script RT Master Mix (2 mL, Takara Bio, Otsu, Japan), mRNA sample (1 μg), and RNase-free ddH_2_O (4 mL) in a total volume of 10 mL. Reactions were set up in triplicate in 96-well plates (20 mL each well) using cDNA (2 mL) with SsoFast Eva Green Supermix (10 mL, Bio-Rad, Hercules, CA, USA), ddH_2_O (7 mL) and gene-specific forward and reverse PCR primers (10 mM, Table 1). The expression of genes encoding collagen type I (COL-1), collagen type II (COL-2), SOX-9, aggrecan (AGN), keratin-18 and keratin-19 was quantified to investigate NPC growth. The housekeeping gene *GAPDH* was used as a control for normalization, and the expression ratio for each marker of interest was determined using the 2^-ΔΔCt^ method. RT-PCR reactions were performed at 95°C for 10 min (activation), followed by 40 cycles amplification (95°C for 10 s, 60°C for 20 s, and 72°C for 20 s), and a final extension (72°C, 1 min), using an ABI Prism 7500 system (Applied Biosystems, Foster City, CA, USA).

**Table 1:** Nucleic acid sequences of forward and reverse PCR primers of specific genes

Gene name	Species	Direction	Sequences
GAPDH	Rabbit	Forward	5′-ATGGTGAAGGTCGGAGTGAAC-3′
GAPDH	Rabbit	Reverse	5′-GTGGGTGGAATCATACTGGAAC-3′
Collagen Type 1	Rabbit	Forward	5′-GAACGGAGATGACGGAGAAG-3′
Collagen Type 1	Rabbit	Reverse	5′-TCCAAACCACTGAAACCTCTG-3′
Collagen Type 2	Rabbit	Forward	5′-GCTCAAGTCCCTCAACAACC-3′
Collagen Type 2	Rabbit	Reverse	5′-CCAGTAGTCACCGCTCTTCC-3′
Sox9	Rabbit	Forward	5′-GGGAAGCTCTGGAGACTGCT-3′
Sox9	Rabbit	Reverse	5′-TGTAGTCCGGGTGGTCTTTC-3′
Keratin 18	Rabbit	Forward	5′-GCTGAAATCGCCACCTACC-3′
Keratin 18	Rabbit	Reverse	5′-TGGTCTTCTGGATGGTCTGC-3′
Keratin 19	Rabbit	Forward	5′-GCTGGCCTACCTGAAGAAGA-3′
Keratin 19	Rabbit	Reverse	5′-GTCAATGCCTGGAGCAGAAT-3′
Aggrecan	Rabbit	Forward	5′-AACAGCCCAAGAAGCAGAAG-3′
Aggrecan	Rabbit	Reverse	5′-GGGTCCAGAAATCCAGAATG-3′

### Second decellularization of SIS particles

After reseeded D-SIS with NPC for 2 months, samples were again decellularized using SDS (1%, Sigma) for 6 h and washed in ultrapure water (12 h). As with the decellularization process (Chapter 5.3), samples were submerged in the decellularization reagent and incubated with agitation (120 rpm).

### Establishment of an IVD degeneration rabbit model

An IVD degeneration rabbit model was induced with a fine needle puncture. [48] The model was confirmed as valid based on previous research describing an MRI signal change within the disc and collapse of disc space. Rabbits were anesthetized with an intravenous injection of pentobarbital sodium (15 mg kg^−1^) and then positioned prone on a platform. Rabbit spines were exposed using a lateral retroperitoneal approach with aseptic techniques. L4-5 and 3–4 IVD were punctured with an 18-gauge needle at a depth of 5 mm and the incision was closed. L5-6 disc was used as a blank control. After the operation, rabbits were individually housed with free access to food and water.

### Injection of DD-SIS microparticles

DD-SIS were collected and suspended in sterile normal saline at a concentration of 50 mg/mL. Injections were performed one month after the IVD puncture. Rabbits (8 rabbits, all male, 2 months of age, weighted 2.2–2.8 kg, mean: 2.64 kg) were anesthetized with an intravenous injection of 15 mg/kg pentobarbital sodium, and spines were exposed using a lateral retroperitoneal approach on the other side. Using a 28-g needle, the IVD of L4-5 and L3-4 were injected with DD-SIS suspension (50 mL) and normal saline, respectively. The small needle size was selected to avoid promoting further degeneration. After surgery, rabbits were individually housed with free access to food and water.

### MRI and X-ray evaluation

Lumbar MRI was performed before the operation and at 0, 1, 2 and 3 months postoperatively, with a GE Sigma CV/I (1.5T) using a knee-joint surface coil. Sagittal T2-weighted images were obtained using repetition/echo times of 2500/120 ms, bandwidth of 31.25 Hz/Px and echo train length of 17, whereas T1-weighted used minifull/550 ms, 31.25 Hz/Px, and echo train length of 2. A central sagittal image of the lumbar spine was selected as a locating image for the next T2 FSE cross-sectional scans at 3000/102 ms and 31.25 Hz/Px and echo train length of 17. The matrix size for each image was the same (matrix size, 384 × 256; field of view, 20 × 20 mm; slice thickness, 2 mm; interslice gap, 0.2 mm). Image J software 1.46 (National Institutes of Health, http://rsbweb.nih.gov/ij/download.html) was used to measure signal intensity of IVD of L3-4, L4-5, and L5-6 on T2-weighted central images. The signal intensity of cerebrospinal fluid was used as contrast. IVD height was also measured using X-ray analysis, calculated as the disc height index (DHI), which indicates the average height of the anterior, middle and posterior disc height. The change in DHI was calculated using a DHI of 0 month as contrast.

### Statistical analysis

Quantitative data are expressed as mean ± standard deviation (STD). Homogeneity test of variance were performed by Levene test, then analysis of variance (ANOVA) was used to compare mean data among groups, with differences between groups analyzed using the least significant difference (LSD) post-hoc analysis. Statistical analyses were performed using SPSS 16.0 (SPSS Inc., Chicago, IL, USA). *P* < 0.05 was considered significant.

## CONCLUSIONS

In summary, we prepared SIS particles by decellularization, modified the SIS via NPC repopulation to make it suitable for IVD regeneration, and decellularized the material again before injection into a rabbit IDD model. The effect of inhibiting intervertebral disc degeneration progression was confirmed *in vivo*. This study showed that a xenogeneic decellularized SIS scaffold has potential therapeutic value for treating intervertebral disc degeneration.
